# Deaf readers benefit from lexical feedback during orthographic processing

**DOI:** 10.1038/s41598-019-48702-3

**Published:** 2019-08-23

**Authors:** Eva Gutierrez-Sigut, Marta Vergara-Martínez, Manuel Perea

**Affiliations:** 1 Department of Psychology, University of Essex, Essex, UK; 2 ERI-Lectura, University of Valencia, Valencia, Spain; 3 UCL DCAL Centre, University College London, London, UK; 4 Nebrija University, Madrid, Spain; 5 Basque Center of Cognition, Brain, and Language, Donostia, Spain

**Keywords:** Language, Reading

## Abstract

It has been proposed that poor reading abilities in deaf readers might be related to weak connections between the orthographic and lexical-semantic levels of processing. Here we used event related potentials (ERPs), known for their excellent time resolution, to examine whether lexical feedback modulates early orthographic processing. Twenty congenitally deaf readers made lexical decisions to target words and pseudowords. Each of those target stimuli could be preceded by a briefly presented matched-case or mismatched-case identity prime (e.g., ALTAR-ALTAR vs. altar- ALTAR). Results showed an early effect of case overlap at the N/P150 for all targets. Critically, this effect disappeared for words but not for pseudowords, at the N250—an ERP component sensitive to orthographic processing. This dissociation in the effect of case for word and pseudowords targets provides strong evidence of early automatic lexical-semantic feedback modulating orthographic processing in deaf readers. Interestingly, despite the dissociation found in the ERP data, behavioural responses to words still benefited from the physical overlap between prime and target, particularly in less skilled readers and those with less experience with words. Overall, our results support the idea that skilled deaf readers have a stronger connection between the orthographic and the lexical-semantic levels of processing.

## Introduction

Deaf individuals often find reading an extremely difficult task. Most deaf adults only achieve a reading level equivalent to that of a 10-year-old (see e.g. English: ref.^1^; Spanish: ref.^2^), and this may negatively impact their academic achievement and social and emotional well-being^3^. Besides general language skills such as having a large vocabulary or good grammatical knowledge, understanding the meaning of a printed word requires fast and accurate decoding of its orthography and phonology^4^. The poor reading skills of individuals born deaf, who consequently have limited access to the phonology of speech, are often explained in terms of their underspecified print-to-sound mapping and poorer use of spoken phonology (e.g., see ref.^5^). Previous research has typically focused on stablishing under what conditions deaf readers use phonological codes from words (see ref.^6^ for a recent overview) and whether this is related to reading ability. However, in a recent study, Gutierrez-Sigut, Vergara-Martínez, and Perea^6^ found that, unlike in hearing readers, the automatic use of phonological information in deaf readers of Spanish was not related to reading skill.

Another possibility is that reading ability in deaf readers is not only related to phonological processing, but to the strength of the links between the orthographic and lexical-semantic levels of processing. In a sentence reading experiment, Bélanger and Rayner^7^ found that deaf individuals skipped more words, re-read less words and refixated words less often than hearing readers normally do, which suggests that, for a given fixation, deaf readers can extract more information than hearing readers. To explain these findings, Bélanger and Rayner^7^ posited the word-processing efficiency hypothesis, which assumes that skilled deaf readers “have tighter connections between orthography and semantics, but also that they are extremely attuned to the visual-orthographic makeup of words and quickly detect precise word form” (p. 224). However, very little is known about the interplay between orthographic and lexical-semantic processing in the earliest stages of visual word processing in deaf readers and whether the degree to which orthographic processing is modulated by the lexical-semantic representations is related to reading ability.

Here we combined a well-known masked identity priming paradigm with the measurement of event related potentials (ERPs) to track the time course of orthographic processing in deaf readers. Specifically, we investigated to what extent lexical-semantic information modulates the encoding of letter identities and whether this process is related to reading ability.

Prior research on hearing readers has consistently shown that they can rapidly access the abstract letter representations of the word’s characters and their accompanying phonological and lexical representations. Most of these experiments have employed the masked priming technique^8,9^. In a masked priming experiment, participants respond to a target stimulus (e.g., “Is the target a word or not?” [lexical decision]) that is briefly preceded by a prime (around 33–50 ms). To further reduce the visibility of the prime, and hence avoid the contamination from non-automatic processes, the prime is typically preceded by a pattern mask (#####) for 500 ms. Information from the masked prime is thought to be integrated with the target rather than being processed as a separate stimulus^10,11^. A demonstration of fast access to abstract, orthographic representations during word recognition is that a number of behavioural lexical decision experiments have shown similar response times to a target word like ALTAR when preceded by a prime that is both nominally and physically identical (e.g., ALTAR) and when preceded by a prime that is nominally, but not physically identical (e.g., altar). This effect of matched- vs- mismatched case has been consistently found not only in adult skilled readers but also in developing readers^12^ and in readers of non-alphabetic scripts (e.g., Hiragana and Katakana^13^). The brain mechanisms involved in masked identity priming were first described by Dehaene *et al*.^14^, who found significant reduction in brain activity (repetition suppression) in a frontal-occipital-temporal network for targets preceded by masked identity primes. Specifically, Dehaene *et al*.^14^ found two right-extrastriate regions where repetition suppression was restricted to matched-case pairs. Furthermore, the left fusiform gyrus and both right and left precentral gyrus showed repetition suppression for masked identity priming independently of case overlap between prime and target (see also ref.^15^ for a review).

Taken in isolation, these findings could be taken as evidence for a purely bottom-up process in which the different allographs of each letter are quickly mapped onto abstract orthographic and phonological representations (e.g., see ref.^16^, for a bottom-up model of letter recognition). However, the study of the response times to pseudowords in lexical decision allows for a different interpretation. Response times to a pseudoword like CURDE are faster when preceded by a nominally and physically identical prime (e.g., CURDE) than when preceded by a prime that is nominally, but not physically identical (e.g., curde)^17–19^. A pure bottom-up model in which the visual input is mapped onto abstract representations cannot explain this finding. Thus, the lack of differences in response times between word pairs like altar-ALTAR and ALTAR-ALTAR can be taken as evidence of lexical-semantic feedback^20^ that overrides the effects of visual overlap in lexical decision. Consistent with this view, when using the masked priming paradigm with a task that primarily taps prelexical processes (i.e., same-different task), Perea, Marcet, and Vergara-Martínez^21^ found an advantage of nominally and physically identical priming condition over the nominally identical priming condition for both words and pseudowords.

Besides the insights provided by behavioural experiments, the measurement of the ERPs time-locked to the target onset allows us to carefully track the time course of visual word recognition (including perceptual, orthographic and lexical stages; see^10^ for a review of the key ERP components for each stage). In a recent ERP lexical decision experiment, Vergara-Martínez *et al*.^19^ found that the dissociation of matched- vs. mismatched-case pairs for words and pseudowords occurred initially at the N250 component, which reflects orthographic processing. The ERPs of word pairs like altar-ALTAR and ALTAR-ALTAR were virtually the same after 200 ms post-target (i.e., once passed the initial perceptual, prelexical stages of word processing), whereas the ERPs of pseudoword pairs like curde-CURDE and CURDE-CURDE differed not only in the initial perceptual time window (N/P150), but also later in processing (in the N250 and N400 time windows).

Interestingly, a recent behavioural masked priming lexical decision experiment with deaf readers^22^ failed to find this dissociation. Results showed faster response times to the target stimuli when preceded by a nominally and physically identity prime than when preceded by a nominally identity prime for both word and pseudoword targets. Perea *et al*.^22^ argued that the lexical-semantic feedback that helps override the purely physical differences in nominally identical pairs in lexical decision is weaker in deaf than in hearing readers. This weaker top-down feedback could reflect an impoverished encoding of phonological information that may hinder the creation of stable orthographic representations. However, neither the participants’ phonological processing nor their reading abilities were measured in that experiment. In the current masked priming lexical decision experiment, we took advantage of the exquisite sensitivity and temporal resolution of the ERPs to characterize the early stages of orthographic processing in deaf readers (via matched-case vs. mismatched-case identity pairs). Furthermore, we also examined whether the obtained effects were modulated by reading skill. The rationale is the following: the lack of an identity masked priming effect of case for words (in contrast to pseudowords) is thought to be due to top-down lexical-semantic feedback, which counteracts facilitation from physical overlap. Importantly, this lexical-semantic feedback depends on the strength of the connection between the orthographic and the lexical-semantic levels, and thus it shall be modulated by reading ability (see e.g. ref.^23^ for an extended explanation). Therefore, if skilled deaf readers have tighter top-down lexical influences than less-skilled deaf readers (as proposed by the word-processing efficiency hypothesis^7^), the effects of case for words (altar-ALTAR vs. ALTAR-ALTAR) would diminish for the better readers—reflecting larger lexical-semantic feedback.

Regarding the ERPs, we focused on the N/P150 and the N250 ERP components because they allow for the dissociation of physical-feature overlap effects from abstract-orthographic overlap effects—for consistency with previous research we also tracked down the effects in a later time window (N400). The N/P150 is sensitive to physical-feature overlap between prime and target but it is not modulated by abstract-orthographic manipulations. For instance, prime-target pairs printed in the same font elicit larger N/P150 amplitudes than pairs printed in different size or font (e.g. table-table > table-table^24^). The N250 is the earliest component sensitive to the degree of orthographic overlap between prime and target, showing larger amplitudes for unrelated pairs than for those with high orthographic overlap (e.g. porch-TABLE > teble-TABLE > table-TABLE^25,26^). Evidence that access to abstract orthographic representations has been achieved by the time of the N250 comes from findings that the N250 amplitude is no longer sensitive to size, shape^24^ nor location of the prime^27^. In relation to this, it has been proposed^14^ that the N/P150 could be the electrophysiological signature of occipital (extrastriate) cortex activity, while the N250 reflects the left fusiform and precentral cortex activity. Critical for the present study, a number of experiments have shown that the N250 is modulated by feedback from higher level processing^19,28,29^.

For the current experiment, as in the Vergara-Martinez *et al*.^19^ study, participants performed a lexical decision task on uppercase targets that were preceded by either a mismatched-case (lowercase) or a matched-case (uppercase) identity masked prime (words: altar-ALTAR vs. ALTAR-ALTAR; pseudowords: curde-CURDE vs. CURDE-CURDE). Regarding early perceptual effects, and according to previous results^10,19^, we predicted that the N/P150 would be sensitive to the match-mismatch case manipulation—rather than to abstract orthographic/lexical information, reflecting the occipital (extrastriate) activity previously observed for perceptual prime-target match in neuroimaging studies^14,15^. Specifically, as this early ERP components is not modulated by abstract-orthographic manipulations, we predicted larger N/P150 amplitudes for the matched than for the mismatched case pairs across both target types (words and pseudowords).

The next relevant component for this study is the N250, which reflects initial mapping to orthographic representations^10^ and has been linked to left fusiform gyrus activity when abstract identity of the letter string has been extracted^14,15,20^. Assuming that deaf readers establish connections between orthographic and lexical-semantic representations as hearing readers do, we predicted the N250 to be sensitive to the match-mismatch case manipulation in pseudoword targets only (not in word targets). Furthermore, if deaf readers have indeed tight connections between orthographic and lexical-semantic representations, we would expect that the magnitude of the ERP effect of match-mismatch case would be similar to that previously observed in hearing readers. To test this question, we contrasted the present data with the previously published data from hearing readers (ref.^19^). Alternatively, if the effect of match-mismatch case remains significant for both words and pseudowords at the N250 time window, this would indicate that, at this processing stage, lexical feedback is not strong enough to counteract visual-perceptual information processing during orthographic encoding in deaf readers (see ref.^22^).

Finally, to test the hypothesis that a tight connection between the orthographic and lexical-semantic levels is related with reading skill in the deaf, we examined the relation between the size of the effect of case for words and the participants’ reading ability (i.e., word knowledge, sentence comprehension, and reading comprehension). If, as proposed by Bélanger and Rayner’s^7^ word-processing efficiency account, more skilled deaf readers have a tighter orthographic-semantic connection, we would expect a correlation between reading ability and the size of the effect of case for words, but not for pseudowords—note that pseudowords lack a lexical-semantic representation. As in the Vergara-Martínez *et al*.^19^ experiment, we also included an unrelated condition. The statistical analyses of the repetition priming effect are shown in the Supplementary Material (Appendix c).

## Results

### Behavioural results

We excluded from the analyses a small subset of stimuli with more than 50% of errors (words: cerco, lecho, rigor, tribu, vejez, verja, verso; pseudowords: argil, fluda, zapio, veter, taror, silar). Incorrect responses (6.5%) and lexical decision times above and below the 2.5 SDs of the average per participant and condition (2.8%) were excluded from the latency analysis. The mean lexical decision times and percentage of correct responses per condition are displayed in Table 1. ANOVAs with the factors Lexicality (words vs. pseudowords) and Case (matched vs. mismatched) were performed separately for the latency and accuracy data (subjects [*F*1] and items [*F*2] analyses were performed for both). The dummy factor List (1–4) was also included in the analyses to extract the error variance due to the counterbalanced lists^30^.

**Table 1:** Mean lexical decision times (RTs, in milliseconds) and percentage of accurate responses for the matched-case, the mismatched-case and the unrelated priming conditions for words and pseudowords in deaf readers.

	Words	Pseudowords
**RT Mean (SD)**		
Matched Case	664 (121)	826 (169)
Mismatched Case	679 (136)	853 (155)
**difference**	**−15**	**−27**
Unrelated	756 (148)	879 (163)
**Accuracy Mean (SD)**		
Matched Case	90.8% (4.8)	95.5% (4.7)
Mismatched Case	92.1% (2.7)	93.4% (8.03)
**difference**	**1.3**	**−2.1**
Unrelated	84.5 (1.2)	94 (1.7)

The latency analyses showed that words were responded to faster than pseudowords (672 vs. 839 ms), *F1*(1,16) = 42.94, *p* < 0.001; *F2*(1,302) = 323.4, *p* < 0.001. In addition, the target stimuli were responded to faster when preceded by a matched-case prime than when preceded by a mismatched case prime (744 vs. 766 ms), *F1*(1,16) = 5.66, *p* = 0.030; *F2*(1, 302) = 9.57, *p* = 0.002. The interaction between Lexicality and Case did not approach significance, both *F*s < 1.

The ANOVA on the accuracy data showed that participants responded more accurately to pseudowords than to words (91.5 vs. 94.5% respectively), *F1*(1,16) = 17.67, *p* = 0.001; *F2*(1, 302) = 1.07, *p* = 0.303. The main effect of Case was not significant, both *F*s < 1. The interaction between the two factors was not significant either, *F1*(1,16) = 2.55, *p* > 0.1; *F2*(1, 302) = 3.38, *p* = 0.067.

### ERP results

**Figure 1:**
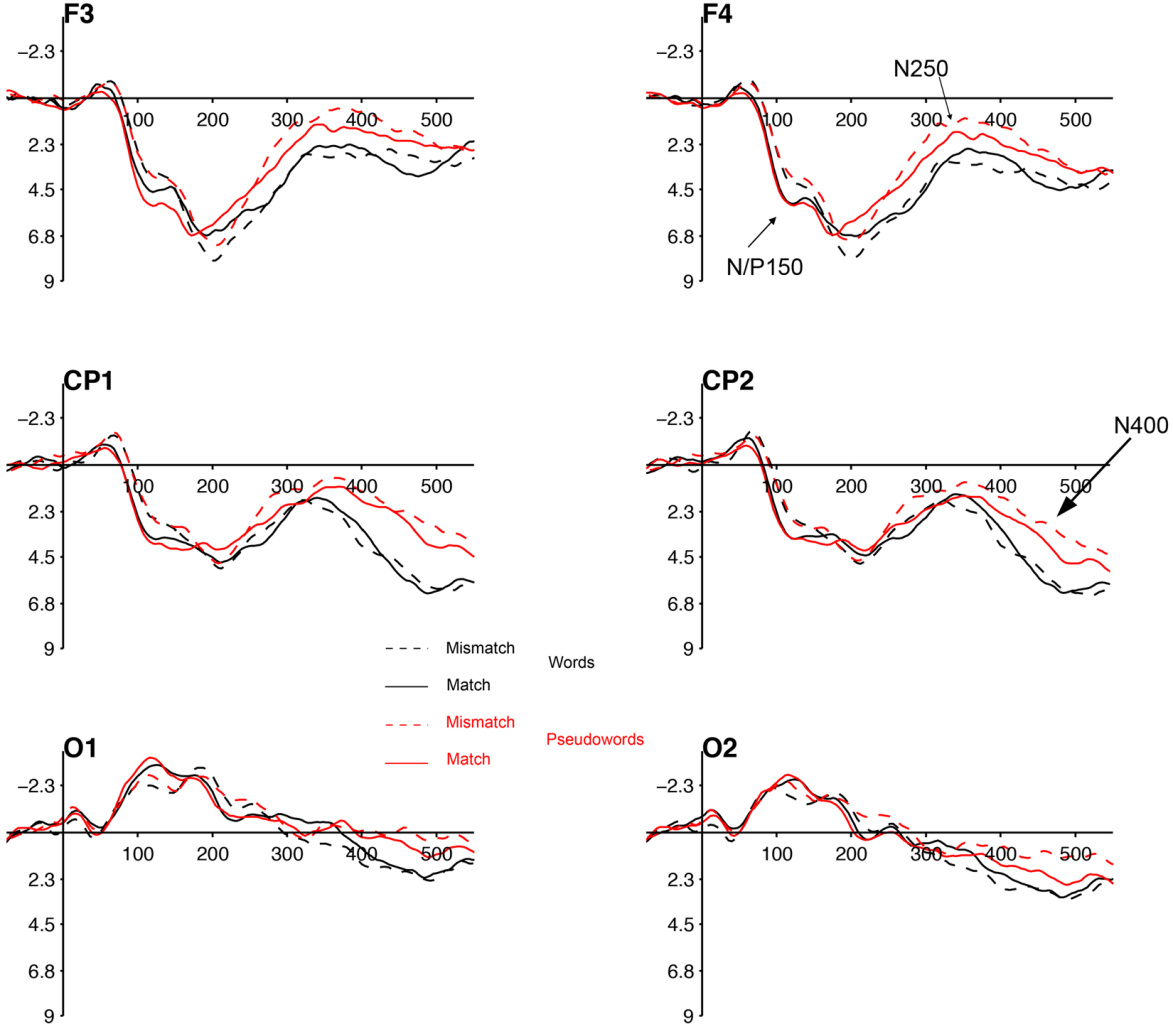
ERP waves. Grand average event related potentials to words (black) and pseudowords (red) in the matched (solid lines) and mismatched-case (dashed lines) conditions in four representative electrodes from the four areas of interest. Electrodes O1 and O2 are displayed at the bottom to illustrate the mirror nature of the N/P150.

**Figure 2:**
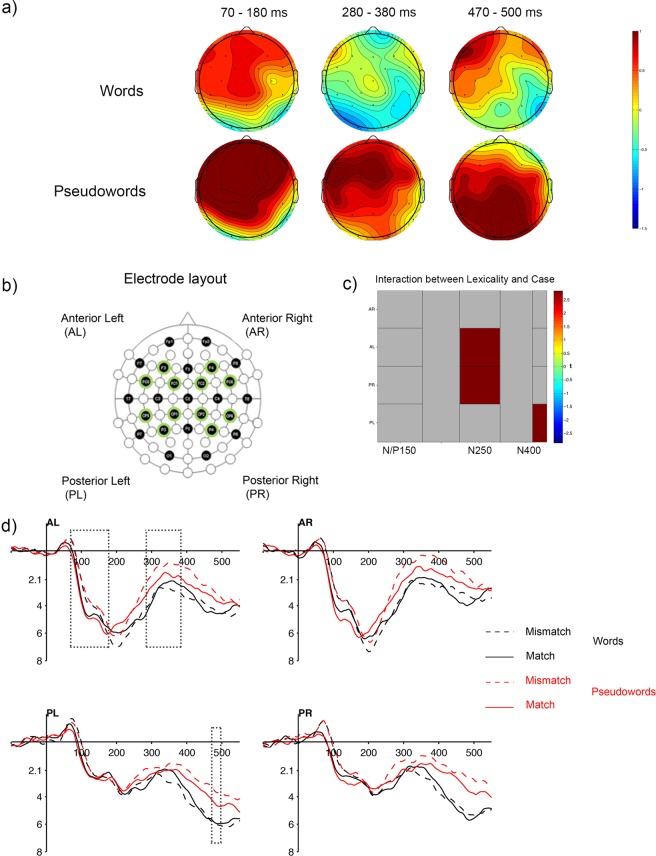
ERP results. Panel (a) shows the scalp maps for the analysed time windows. Panel (b) shows a schematic representation of the electrode montage. Electrodes are grouped in four different areas (anterior-left, anterior-right, posterior-left and posterior-right) for statistical analyses. Panel (c) displays the interaction between lexicality and case at the four regions of interest for the 3 large windows analysed (inputting the difference of the difference waves in the Mass Univariate ERP toolbox). Consistently with results from the ANOVAs, the figure shows a significant interaction between the two variables at the later time windows, but not the first. Panel (d) depicts the ERP waves at the four regions of interest.

Figure 1 shows the ERP waves of the matched-case and mismatched-case conditions for the words (black) and pseudowords (red) in four representative electrodes—the occipital electrodes are also displayed to illustrate the bipolar nature of the N/P150 component. In addition, Fig. 2 (panel d) shows the ERP waves for the experimental conditions in the four groups of electrodes included in the analyses. The ERPs in the target epoch produced an initial negative component peaking around 50 ms, which was followed by a larger and slower positivity (P2), larger at anterior areas and ranging between 90 and 280 ms (this pattern is opposite in occipital electrodes). Following these early potentials, a large and slow negativity peaking around 350 ms can be seen at both anterior and posterior areas.

To inform the selection of three time-windows reflecting the N/P150, N250, and N400 components, respectively, we performed a univariate analysis (see EEG recording and analyses section for details and Appendix A for results). Results of this analysis revealed, in essence, significant differences between matched- and mismatched-case targets, consistent with the N/P150 component, between approximately 70 and 180 ms at anterior and central electrodes bilaterally. Further significant differences were found for pseudoword targets consistent with the timings and distribution of the N250 (280 to 380 ms) and the N400 (470 to 500 ms) components. Based on these analyses, repeated measures ANOVA including the factors Lexicality and Case were performed separately on each of the following time windows: 70–180 ms, 280–380 ms and 470–500 ms (see also Fig. 2, panels a and c for further evidence regarding the differences in distribution that support the differentiation of the N250 and N400 components).

#### 70–180 ms

The statistical analyses showed a main effect of Case, *F*(1,16) = 8.51, *p* = 0.010, which reflected larger positive amplitude values for the matched–case condition than the mismatched-case. There were no signs of an effect of Lexicality or an interaction between lexicality and case, both *F*s < 1.

The interaction between Case and A-P distribution was significant, *F*(1,16) = 5.40, *p* = 0.033: The effect of case was larger in anterior (*F*(1,16) = 10.82, *p* = 0.005) than in posterior (*F*(1,16) = 4.54, *p* = 0.049) electrode sites. The three-way interaction between Case, A-P distribution and hemisphere was also significant, *F*(1,16) = 4.49, *p* = 0.050. The effect of case was significant at left anterior (*F*(1,16) = 8.22, *p* = 0.002), right anterior (*F*(1,16) = 10.29, *p* = 0.011) and right posterior (*F*(1,16) = 7.46, *p* = 0.015) but not at left posterior (*F*(1,16) = 2.12, *p* > 0.1) electrode sites. The other interactions did not approach significance (all *p*s > 0.3).

#### 280–380 ms

The main effect of Lexicality was significant, *F*(1,16) = 15.22, *p* = 0.001. The main effect of Case, *F*(1,16) < 1, was not significant. The interaction between lexicality and A-P distribution, *F*(1,16) = 9.92, *p* = 0.006, which revealed that the lexicality effect was stronger over anterior (*F*(1,16) = 24.9, *p* < 0.001) than posterior (*F*(1,16) = 6.35, *p* = 0.023) electrode sites.

The interaction between lexicality and case was significant, *F*(1,16) = 4.7, *p* = 0.046. This interaction showed an effect of case for pseudoword targets (*F*(1,16) = 7.54, *p* = 0.014), but not for word targets (*F* < 1). There were no other significant interactions (all *p*s > 0.15).

#### 470–500 ms

There was a significant main effect of Lexicality, *F*(1,16) = 11.42, *p* = 0.004. The main effect of Case was not significant, *F*(1,16) = 3.1, *p* = 0.097. The interaction between lexicality and A-P distribution, *F*(1,16) = 5.44, *p* = 0.033, showed that, unlike the previous window, the lexicality effect was stronger over posterior (*F*(1,16) = 11.94, *p* < 0.003) than anterior (*F*(1,16) = 7.67, *p* = 0.014) electrode sites.

The interaction between lexicality, case and A-P distribution was also significant, *F*(1,16) = 6.12, *p* = 0.025: the effect of case was significant only for pseudoword targets at posterior sites (*p* = 0.041; other *p*s > 0.1). There were no other significant interactions (all *p*s > 0.19).

### Comparison with previously published ERP data from hearing readers

For comparability with the previously reported data from hearing readers we performed the same ANOVAs on the following time windows: 80–150 ms, 250–350 ms and 400–500 ms.

#### 80–150 ms

The statistical analyses showed a main effect of Case, *F*(1,16) = 10.996, *p* = 0.004, which reflected larger positive amplitude values for the matched–case than the mismatched-case condition. There were no signs of an effect of Lexicality or an interaction between lexicality and case, both *F*s < 1.

The interaction between Case and A-P distribution was significant, *F*(1,16) = 5.16, *p* = 0.037: The effect of case was larger in anterior (*F*(1,16) = 12.85, *p* = 0.002) than in posterior (*F*(1,16) = 6.15, *p* = 0.025) electrode sites. The other interactions did not approach significance (all *p*s > 0.1).

#### 250–350 ms

The main effect of Lexicality was significant, *F*(1,16) = 19.89, *p* < 0.000. However, the main effect of Case, *F*(1,16) = 2.39, *p* < 0.142, was not significant. The interaction between lexicality and A-P distribution was significant, *F*(1,16) = 11.23, *p* = 0.004, which revealed that the lexicality effect was stronger over anterior (*F*(1,16) = 35.7, *p* < 0.001) than posterior (*F*(1,16) = 6.04, *p* = 0.026) electrode sites. The interaction between lexicality and case did not reach significance, *F*(1,16) = 1.72, *p* = 0.209. There were no other significant interactions (all *p*s > 0.15).

#### 400–500 ms

Only the lexicality effect was significant, F(1,16) = 15.95, p = 0.001 (remaining *p*s > 0.1).

In order to test whether the magnitude of the ERP effect of case (difference between the amplitudes in the matched and mismatched case conditions) is the same for deaf and hearing readers, we contrasted the present data with the previously published data from hearing readers^19^. We performed ANOVAs on the size of the effect of case including the within-subjects factors hemisphere, A-P distribution and Lexicality (words vs. pseudowords) and the between-subjects factor Group (deaf vs. hearing readers). In all analyses, List (1–4) was included as a dummy between-subjects factor. A separate ANOVA was run for each of the identified components (N/P150, N250 and N400), as we use the time windows previously identified for the data from the hearing group (see Table 2 for details; for analysis using time windows appropriately adapted to each dataset see Supplementary Material Appendix B).

**Table 2:** Comparison with previously published data from hearing readers.

	df	N/P150		N250		N400	
		*F*	*p*	*F*	*p*	*F*	*p*
AP	(1,34)	<1		2.95	0.095	8.05	0.008
Hem	(1,34)	17.08	0.000	10.61	0.003	9.94	0.003
Lexical	(1,34)	1.4	0.244	8.69	0.006	6.69	0.014
AP * Hem	(1,34)	26.2	0.000	8.265	0.007	9.33	0.004
AP * Lexical	(1,34)	11.81	0.002	13.62	0.001	19.45	0.000
Hem * Lexical	(1,34)	15.59	0.000	15.24	0.000	13.91	0.001
AP * Hem * Lexical	(1,34)	20.77	0.000	9.73	0.004	11.89	0.002
Group	(1,34)	<1		4.28	0.046	1.68	0.203
AP * Group	(1,34)	1.11	0.3	4.45	0.042	<1	
Hem * Group	(1,34)	1.27	0.267	<1		1.79	0.189
Lexical * Group	(1,34)	<1		1.53	0.225	1.76	0.194
AP * Hem * Group	(1,34)	<1		<1		<1	
AP * Lexical * Group	(1,34)	<1		1.33	0.257	<1	
Hem * Lexical * Group	(1,34)	<1		<1		<1	
AP * Hem * Lexical * Group	(1,34)	<1		<1		<1	

Results of the ANOVAs on the size of the effect of case including the within-subjects factors hemisphere, A-P distribution and Lexicality (words vs. pseudowords) and the between-subjects factor Group (deaf vs. hearing readers) for each of the identified.

#### N/P150: 80–150 ms

There were no significant interactions with group (all *p*s > 0.26).

#### N250: 250–350 ms

There was a significant interaction between A-P distribution and group, F(1,34) = 4.5, p = 0.042,: the effect of case was larger for deaf than hearing participants at posterior (F = 5.7, p = 0.023) but not at anterior electrodes (F < 1). There were no other significant interactions with group (all *p*s > 0.22).

#### N400: 400–500 ms

There were no significant interactions with group (all *p*s > 0.18).

### Correlations with reading measures

**Figure 3:**
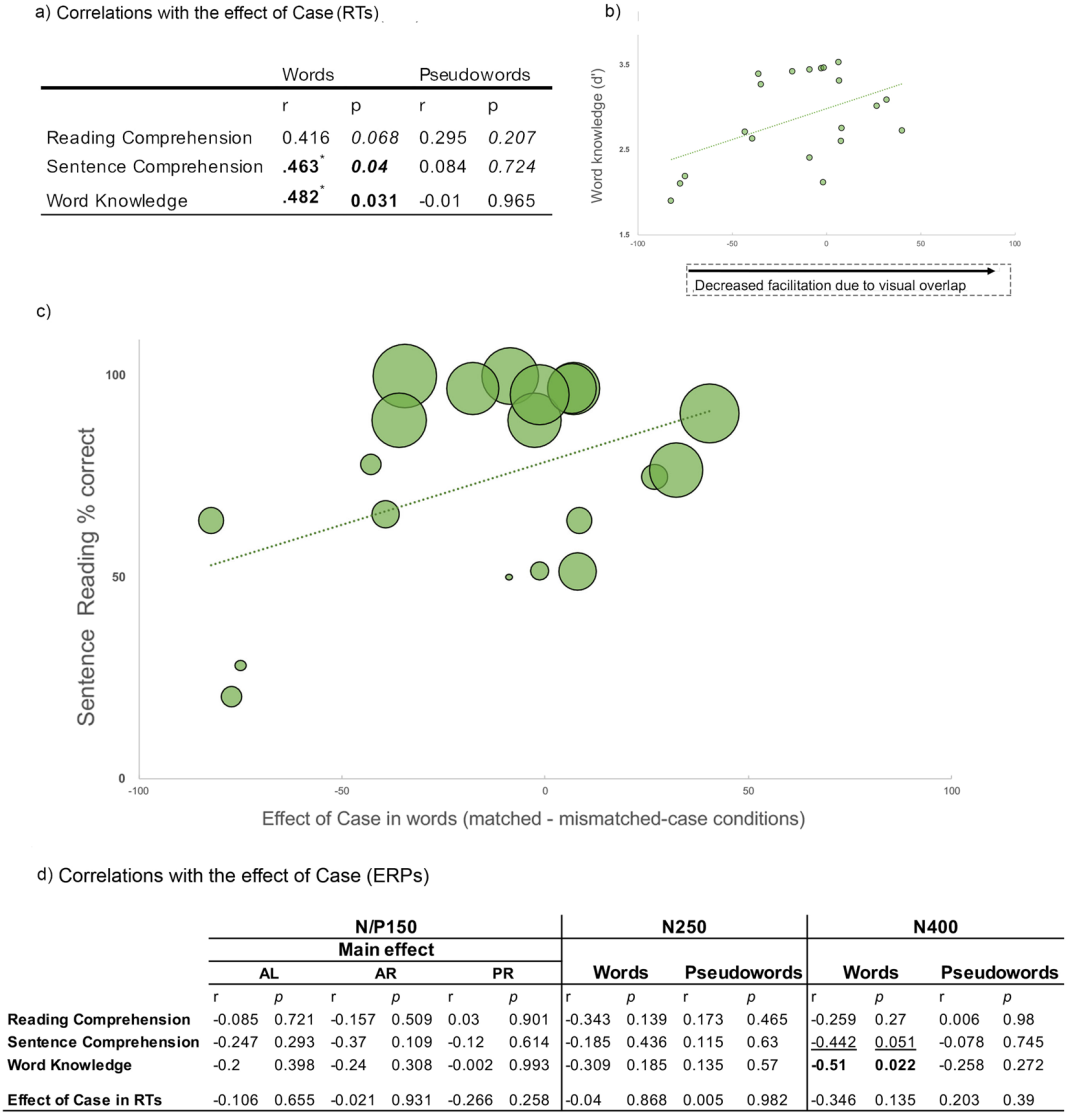
Correlations with behaviour. Panel (a) correlations between the behavioural effect of Case for words and pseudowords separately and reading related variables. Panel (b) relationship between the effect of case and word knowledge. Panel (c) relationship between the effect of case and Sentence Comprehension (the size of the bubble represents reading comprehension score, which is correlated with sentence reading). Panel (d) shows the correlations between the ERP effects of case at the N/P150, N250 and the N400 windows and the reading related-variables (top rows) and response times during the on-line task (bottom rows).

We found no signs of a relationship between the main effect of case in the RTs and the reading variables (all *p*s > 0.5). However, as our predictions involved correlations to occur for word targets only, we examined the correlations for word and pseudoword targets separately. The Pearson correlation coefficients between the size of the effect of case (the difference in response times between matched-case and mismatched-case pairs) and the performance in the reading related measures are shown in Fig. 3 (panel a).

For pseudowords, the behavioural effect of case did not significantly correlate with performance in any of the reading related variables measured (see Fig. 3 panel a). Importantly, for words, the effect of case correlated significantly with sentence comprehension, word knowledge, and orthographic/visual bias during syllabification. Deaf participants with lower reading ability showed a larger effect of case and, similarly, deaf participants with lower word knowledge showed a larger effect of case (see Fig. 3 panels b and c). In other words, less skilled deaf readers were more affected by the physical similarity between the prime and the target words than more skilled deaf readers. In line with these results, deaf readers that were more biased by orthographic/visual factors when performing an explicit phonological task (on-line syllabification) showed more facilitation due to physical overlap between the prime and the target words.

We also examined whether the size of the ERP effect of case (i.e., the difference in the amplitude values between the matched and the mismatched-case conditions) correlated with reading measures. Remember that the ERP effect of case was present for all targets in the N/P150, but only for pseudowords in the N250 and in the N400. We focused the correlational analyses on those electrode clusters where the effect of case was significant. There were no significant correlations between the ERP effects and reading comprehension, sentence reading, word knowledge nor orthographic/visual bias during syllabification for the N/P150 and the N250 time windows. For the N400 window we found a significant correlation between word knowledge and the ERP effect for words. Likewise, the correlation between sentence comprehension and the ERP effect for words approached significance. Similar to the pattern found in the correlational analyses for the RTs, participants with smaller vocabularies showed a larger N400 amplitude difference between matched- and mismatched-case word targets. That is, deaf readers with smaller vocabularies, and to certain extent poorer deaf readers, were more affected by the physical similarity between the prime and the target words than deaf readers with larger vocabularies.

## Discussion

We conducted a masked priming lexical decision experiment to investigate the influence of lexical-semantic feedback on orthographic encoding in deaf readers. Specifically, we examined the time course of the differences between nominally identical prime-target pairs that were physically the same or not (e.g., words: [matched-case] ALTAR-ALTAR vs. [mismatched-case] altar-ALTAR; pseudowords: CURDE-CURDE vs. curde-CURDE). We found a main effect of case in an early component associated to perceptual processes (N/P150). Critically, the difference between matched-case and mismatched-case pairs remained significant for pseudowords but not for words in the components associated to orthographic (N250) and lexical-semantic processes (N400), thus replicating and extending the findings reported by Vergara-Martínez *et al*.^19^ with hearing readers.

Firstly, all target items, regardless of their lexical status, were sensitive to prime-target visual overlap at a very early level of analysis, as shown by the larger amplitudes for the matched-case pairs in the N/P150 component. The N/P150 is a bipolar ERP component characterized by positive amplitude values at anterior electrodes (accompanied by simultaneous negative polarity at posterior sites, see Fig. 1 for an illustration) that reaches its maximum peak between approximately 80 and 200 ms. The timing, the anterior distribution (particularly at left anterior sites; see Supplementary Material, Appendix A for a depiction of the time course of the effect in each electrode) and the direction of the obtained N/P150 effect are consistent with previous ERP masked priming word recognition experiments (see ref.^10^ for a review). The lack of an interaction with lexicality, together with its early timing and distribution, favour the view of the N/P150 component as reflecting initial activation of visual features in occipital cortex in a feedforward manner^14,15,20^—before top down feedback modulates processing. This is, very early during stimulus processing, the system merely evaluates the visual match between prime and target, based on basic low-level feature information.

Secondly, the critical dissociation in the effect of case for words and pseudowords —the differences between matched-case and mismatched-case identity pairs remained for pseudowords but not for words— started at around 280 ms post-target (N250) and had a typical anterior left distribution^10^ (see Supplementary Materials, Appendix A for the effects on each of the electrode sites). As the N250 is typically interpreted as a marker of access to abstract orthographic representations^10,25,31,32^, our results indicate that, in deaf readers—as it has been previously observed in hearing readers, the retrieval of abstract letter identities is influenced by the activation of lexical entries. This finding provides further support to models of visual word recognition that posit fully interactive processes among multiple representation levels. Our results can be interpreted in the context set up by neuroimaging studies that have found involvement of a frontal-occipital-temporal network for written word recognition (see ref.^20^ for a detailed account). For example, Twomey *et al*.^33^ found that the activation of the left ventral-occipito-temporal (VoT) cortex, an area involved in early stages of processing during visual word recognition, was modulated by higher level factors such as the focus on phonological or orthographic information (as specified by the task) during lexical decision. Furthermore, Woodhead *et al*.^34^ found that during the first 200 ms of stimulus processing, words (when compared to false fonts) elicited more activation in the left inferior frontal gyrus. Importantly, they found strong feedback connections from the inferior frontal gyrus to the ventral occipital cortex for word processing (see refs^35–38^ for similar evidence). Consistent with findings of the involvement of this frontal-occipital-temporal network for word recognition in hearing readers, Glezer *et al*.^39^ found selectivity for orthography in both the left inferior frontal gyrus and the left ventral-occipital-temporal cortex in deaf readers.

A comparison of the present ERP data with the previously published data of hearing readers^19^, using time windows previously identified for hearing readers (see Supplementary Materials, Appendix B for the same results using time windows appropriately adapted to each data set), showed that there were no differences in the size of the effect of case for any of the studied components, although the N250 was slightly more widely distributed in deaf readers. This lack of differences in the size of the effect of case from the N250 time window goes in line with behavioural and neuroimaging studies that found similar orthographic effects in deaf and hearing readers despite differences in reading ability^40^. For example, Bélanger, Mayberry, and Rayner^40^ found orthographic preview benefits of similar magnitude in hearing and deaf readers, both skilled and less skilled (see also ref.^41^ for similar transposed-letter effects in hearing and skilled deaf readers). Regarding brain activity during orthographic processing, recent fMRI studies show similar strength of activation of the left ventral occipito-temporal cortex (vOT; a brain area thought to be involved in word and letter identification during the early stages of visual word recognition^42^) for hearing and deaf readers that differ in reading skill^43–45^. Finally, an effect of case of similar size for deaf and hearing readers is also consistent with a recent ERP experiment that found comparable left lateralization of the N170 at occipital electrodes in both populations^46^. Altogether, these findings suggest parallel early orthographic processing for deaf and hearing readers. Nonetheless, the inspection of the time course of the orthographic processing can enrich this picture.

Interestingly, in comparison with the results from hearing readers reported by Vergara-Martínez *et al*.^19^, we found slight differences in the time-course of the effect of case for words and pseudowords (N250 and N400). Specifically, the N250 effect was somewhat delayed for deaf when compared to hearing readers, and the N400 effect was remarkably short in the current experiment. In line with this, the interaction between case and lexicality was not significant in deaf readers for the slightly earlier N250 and the wider N400 time windows used for hearing readers. We acknowledge the possibility that these time-course differences could be due to the data-driven approach used in the time-epoch definition in the present study. However, these timing differences were only found in the effect of case, but not in the repetition priming effect (mismatched-case identity condition vs. unrelated; see Supplementary Materials, Appendix c). Indeed, the N250 repetition priming effect started as early as 200 ms after target onset and the N400 was present for the whole 400 to 490 ms window. These timings are very similar to those reported by the Vergara-Martinez *et al*.^19^ repetition priming data (Appendix B, Fig.S5). Thus, the differences in the effect of case between deaf and hearing are unlikely due to the analysis methods.

The delay in the N250 and the short N400 effects found here may reflect that the lexical feedback for deaf readers, although present, was slightly weaker than for hearing readers. Interactive models of word processing posit that the initial feedforward neural activity elicited by a printed word is soon stabilized by reactive feedback from higher levels of processing (i.e. orthographic, phonological, lexical-semantic; see refs^10,20^). Recently, Dufau *et al*.^32^ proposed that this reactive feedback^10,20^ interacts with proactive feedback mechanisms, and that differences in proactive feedback can result in differences in the time course of word processing (see e.g. Strijkers *et al*.^47^ for differences in the time course of lexical frequency depending on the linguistic vs. perceptual focus of the task). In accordance with this view, Emmorey *et al*.^46^ argued that early differences in P1 amplitude between deaf and hearing readers could be explained in terms of differential top-down feedback: either attentional or reactive feedback mechanisms (due to altered visual experience and to atypical acquisition of reading respectively) can result in differential top-down feedback in deaf readers. Similarly, the slightly delayed N250 found here could be interpreted as a reflection of subtle differences in feedback mechanisms in deaf readers.

Consistent with this explanation, one notable finding of the present experiment is that the dissociation between words and pseudowords for the matched-case manipulation found in the ERPs (N250 and N400) was not reflected in the behavioural data. That is, the matched-case manipulation did have an impact on behavioural responses to both words and pseudowords. These behavioural findings replicate those of Perea *et al*.^22^ in a similar experiment with another group of deaf readers and another set of items. Remarkably, despite the parallelisms in the ERP outcomes of both Vergara-Martinez *et al*.^19^ and the present studies, the behavioural results differed. In the Vergara-Martinez *et al*.^19^ study with hearing readers, the behavioural results mirrored the ERP effects found in the N250 and N400 components: the effect of case was present for pseudowords, but not for words. However, in the present study with deaf participants, the behavioural responses mirrored the ERP effects found in the N/P150, an early perceptual ERP component, where case effects were present for all targets. In other words, deaf readers’ behavioural responses to word targets seemed to still benefit from the physical overlap between prime and target regardless of the electrophysiological evidence of lexical-semantic feedback during abstract orthographic encoding. This behavioural pattern of data adds further support to the proposal that lexical feedback might not be strong enough to completely counteract the perceptual relation between prime and target (effect of case) in deaf readers. The results from the correlational analyses can shed further light on this issue: the size of the behavioural effect of case for words correlated with reading skill and word knowledge. Specifically, the effect of case was smaller for those deaf readers with better reading skill, larger word knowledge, and better phonological abilities. This pattern of correlations suggests that, as proposed by the word-processing efficiency hypothesis^7^, lexical feedback modulated orthographic processing to a larger degree in skilled than in less skilled deaf readers. In light of the present results, it is possible that the lexical feedback, although present—as it can be tracked down with the highly sensitive ERPs measures, is simply not strong enough to feed into the behavioural response, particularly for the less skilled readers.

In sum, our results provide strong evidence of early automatic lexical-semantic feedback modulating the orthographic processing of deaf readers, as demonstrated by the dissociation between words and pseudowords at the time of the N250. The results of the correlational analyses support the word-processing efficiency hypothesis^7^: skilled deaf readers have a stronger connection between the orthographic and the lexical-semantic levels of processing. Thus, one could hypothesise that increasing the experience at recognizing printed words (word knowledge) would contribute to strengthen the lexical feedback and hence the link between the orthographic form and word meaning, resulting in better reading ability.

## Methods

### Participants

Twenty congenitally deaf (note that Deaf—as opposed to deaf—is often used to refer to deaf people who use sign language and are part of the Deaf community. Although all our participants are Deaf, we use the term deaf through this manuscript to refer to their hearing status.) participants (8 female) participated in this experiment. All participants were profoundly deaf, skilled signers, right-handed, had no history of neurological or psychiatric impairment, and had normal (or corrected-to-normal) vision. Seven participants were native signers of Spanish Sign Language (LSE [Lengua de Signos Española]), eight were early signers (learn sign language before the age of 9) and five were late signers. None of the effects reported here (behavioural or ERPs) correlated with age of SL acquisition, all *p*s > 0.141. Their ages ranged from 21 to 56 years (M = 36.4, SD = 9.3).

All participants were tested on three reading-related measures (see ref.^6^ for detailed descriptions of these tests): (1) reading comprehension (M = 50.7% correct responses, SD = 23.5, range 10–82%; tested with TALE 2000^48^), (2) sentence reading (M = 74% correct responses, SD = 24, range 20–100%; tested with TECLE^49^) and (3) an explicit phonological task (syllable counting; M = 76.9% correct, SD = 11.5, range 49.2–91.0% correct). For the syllable counting task, we computed index of the degree in which orthographic/visual factors (i.e. word length) influenced the participants’ response during an online syllable counting task. This index was obtained as a function of accuracy of responses to highly consistent and highly discrepant words regarding their phonological and orthographic structure. The higher the value of this index the larger the orthographic/visual bias during online syllabification (see ref.^6^ for further details).

In addition, as there were no standardized measures for adults, we used participant’s d’ value for responses to several online lexical decision experiments (over 400 Spanish words and pseudowords in total) as a proxy to word knowledge.

In order to evaluate consistency, we explored the correlations between these reading-related measures: Reading comprehension was highly correlated with sentence reading (*r* = 0.82, *p* < 0.001), word knowledge (*r* = 0.64, *p* = 0.002), and negatively correlated with the orthographic/visual bias during syllabification index (*r* = −0.64, *p* = 0.002). Sentence reading was also highly correlated with word knowledge (*r* = 0.78, *p* < 0.001), and negatively correlated with the orthographic/visual bias during syllabification index (*r* = −0.76, *p* < 0.001), showing that more skilled deaf readers were less biased by visual factors during an explicit phonological task.

This study was approved by the Research Ethics Committee of the University of Valencia, this research was conducted according to the relevant guidelines, and all participants gave written informed consent before the experiment. Information necessary for the informed consent was given to deaf participants both in writing and in LSE.

### Materials

The target stimuli were 160 five-letter Spanish words and 160 matched pseudowords taken from a previous masked priming experiment with Spanish readers^19^ (see appendix A for the full set of stimuli). The target words and pseudowords were presented in uppercase and were preceded by: (a) a matched-case identity prime (ALTAR-ALTAR, matched-case condition); (b) a mismatched-case identity prime (altar-ALTAR, mismatched-case condition), (c) an unrelated word prime (half un lowercase and half in uppercase) and (d) an unrelated pseudoword prime (half un lowercase and half in uppercase). Four counterbalanced lists of materials were constructed in a Latin-square type so that each target appeared once in each list, while all conditions were present in each list.

### Procedure

Participants were seated comfortably in a darkened room. All stimuli were presented on a high-resolution monitor that was positioned slightly below eye level, 85–90 cm in front of the participant. The size of the stimuli and distance from the screen allowed for a visual angle of less than 3.6 degrees horizontally. The procedure was the same than the Vergara-Martínez *et al*.^19^ study except for a 2000 ms extra time for blinking after the target stimulus. Stimuli were presented in the center of the screen, in white 24-pt Courier font against a dark-gray background. The participants viewed a pattern mask (#####) for 500 ms, then the prime for 33.3 ms followed by a 16.7 ms pattern mask and, finally, the target stimulus until the participant responded or 2,500 ms had elapsed. After participants’ response, the drawing of an eye stayed on screen for 2,000 ms to allow for blinks, followed by a blank screen of a random duration between 700 and 1,000 ms. Participants were asked to decide as fast and accurately as possible if the target stimulus was a real Spanish word or not. They pressed one of two response buttons (SÍ [YES]/NO), the hand used for each response was counterbalanced across participants. RTs were measured from target onset until the participant’s response. Each participant was randomly assigned to one of the four counterbalanced lists. The order of stimuli presentation from each list was randomized for each participant. Before the experiment began, participants were given a 16 trials long practice session.

### EEG recording and analysis

The electroencephalogram (EEG) was recorded from 33 Ag/AgCl active electrodes (four of them around the eyes to record the electrooculogram) referenced to the right mastoid. The recording was re-referenced offline to the average of left and right mastoids. Signals were sampled continuously with a sampling rate of 250 Hz, and band-pass filtered offline between 0.01–20 Hz. Initial analysis of the EEG data was performed using the ERPLAB^50^ for EEGLAB^51^. Epochs of 550 ms post-target onset, with a 150 ms baseline were analysed. Trials with eye movements, blinks, muscle activity or other artifacts were rejected (all participants had more than 20 valid trials in each condition—there were no significant differences in the number of rejected trials between conditions, *p*s > 0.3).

To characterize the time course of the effect of case we performed repeated measures, two-tailed t-tests at each sampling point between 50 and 550 ms at 15 scalp electrodes (i.e. F3, FC1, FC5, CP1, CP5, P3, Fz, Cz, Pz, F4, FC2, FC6, CP2, CP6 and P4; total of 1890 comparisons; see Fig. 2, panel a). The Benjamini and Yekutieli^52^ procedure for control of the false discovery rate (FDR: i.e. mean proportion of significant test results that are actually false discoveries or Type I errors) was applied to assess the significance of each test using an FDR level of 5%.

We tested the main effect of case by comparing the matched and mismatched-case stimuli (including all targets). Then, as we predicted that the effect would remain significant in later time windows for pseudoword targets only, we compared matched and mismatched-case in pseudoword targets separately. The pattern of results found in this univariate analysis, that provides good temporal resolution and maintains reasonable limits on the likelihood of false discoveries, informed the selection of the 3 time windows that allowed for assessment of the N/P 150, N250 and N400 components respectively: 70–180 ms, 280–380 ms, and 470–500 ms (see Fig. 2; see also ref.^53^ for a similar data-driven approach to finely tune the typically used a priory time windows). The repetition priming effect (identity vs. unrelated conditions) was submitted to parallel repeated measures t-tests (see Supplementary Material, Appendix C).

The univariate analysis was used in lieu of separate analysis of variance (ANOVA) for each selected time window. The ANOVAs included the within subjects factors hemisphere, A-P distribution Case (match vs. mismatch) and Lexicality (words vs. pseudowords).To analyse the topographical distribution of the effects we included the averaged amplitude values across three electrodes in four representative scalp areas that resulted from the factorial combination of the factors hemisphere (left vs. right) and anterior-posterior (A-P) distribution (anterior vs. posterior): anterior left (AL: F3, FC1, FC5), anterior right (AR: F4, FC2, FC6), posterior left (PL: CP1, CP5, P3) and posterior right (PR: CP2, CP6, P4). In all analyses, List (1–4) was included as a dummy between-subjects factor in order to extract the variance that was due to the counterbalanced lists^30^. Main effects of lexicality are reported when they are relevant for the interpretation of the results. Effects of hemisphere or A-P distribution factors are only reported when they interact with the experimental manipulations. Interactions between factors were followed up with simple-effects tests. For comparability with findings in hearing participants we performed the same ANOVAs on the windows previously reported for hearing readers. Furthermore, we tested whether the magnitude of the ERP effect of case was similar for deaf and hearing readers by contrasting the present data with the previously published data from hearing participants^19^.

## Supplementary information

Deaf readers benefit from lexical feedback during orthographic processing

## Data Availability

The datasets generated during and/or analysed for the current study are available from the corresponding author.
