# Supplementary material for: Deaf readers benefit from lexical feedback during orthographic processing
Source: Sci Rep. 2019 Aug 23;9:12321. doi: 10.1038/s41598-019-48702-3 (PMC6707270; doi:10.1038/s41598-019-48702-3)
Supplement: Supplementary file 1 — Deaf readers benefit from lexical feedback during orthographic processing [file 41598_2019_48702_MOESM1_ESM.pdf]

Supplementary information file

Deaf readers benefit from lexical feedback during orthographic processing

*Eva Gutierrez-Sigut[1,2], Marta Vergara-Martínez[1], Manuel Perea[1, 3]

[1] ERI-Lectura, University of Valencia, Spain
[2] UCL DCAL Centre, University College London, UK
[3] Nebrija University, Madrid, Spain

Supplementary material: Appendix A. Details of the Mass Univariate Analyses for the effect of case

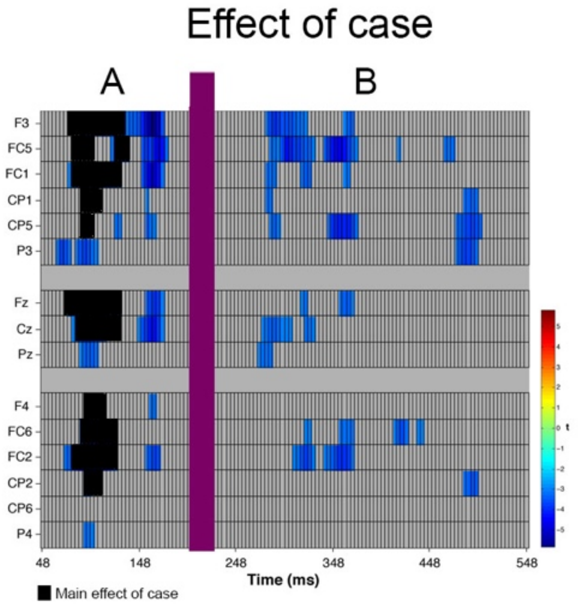

Supplementary figure S4. Time points and electrodes where a significant difference between matched- and mismatched-case was found. **A** depicts the results of the mass univariate statistical analysis of the time course of the main effect of case (black) overlapped to the results for pseudoword (blue) targets separately between 48 and 200 ms. **B** shows the results of the mass univariate statistical analysis of the time course of effect of case for pseudoword targets between 200 and 550 ms.

Supplementary table 2. Detailed results of the Mass Univariate Analyses of the effect of case

Analysis parameters

Time boundaries of 50 to 550 ms for hypothesis test (exact window boundaries 48 to 548 ms).	Number of channels: 15. Number of time points: 126. Total # of comparisons: 1890	Number of participants: 20. t-score degrees of freedom: 19	FDR control procedure: Benjamini & Hochberg (independent or positive dependency)

**ERP component**<br>**Comparison**	**ms**	**N/P150**<br>**electrodes with significant differences**	**ms**	**N250**<br>**electrodes with significant differences**	**ms**	**N400**<br>**electrodes with significant differences**
**Main effect of case**	**72**	Fz	**200**	F4	**476**	FC5
	**76**	Fz, F3	**204**	F4, FC2	**480**	FC5
	**80**	Fz, F3, FC5, FC1, FC2	**208**	F4, FC2	**484**	FC5, CP5
	**84**	Fz, Cz, F3, FC5, FC1, FC2	**212**	F4, FC2	**488**	FC5, CP5
	**88**	Fz, Cz, F3, FC5, FC1, FC2, FC6, CP5, CP1			**492**	FC5, CP5
	**92**	Fz, Cz, F3, F4, FC5, FC1, FC2, FC6, CP5, CP1, CP2			**496**	CP5
	**96**	Fz, Cz, F3, F4, FC5, FC1, FC2, FC6, CP5, CP1, CP2				
	**100**	Fz, Cz, F3, F4, FC5, FC1, FC2, FC6, CP5, CP1, CP2				
	**104**	Fz, Cz, F3, F4, FC1, FC2, FC6, CP1, CP2				
	**108**	Fz, Cz, F3, F4, FC1, FC2, FC6, CP1, CP2				
	**112**	Fz, Cz, F3, F4, FC1, FC2, FC6				
	**116**	Fz, Cz, F3, FC1, FC2, FC6				
	**120**	Fz, Cz, F3, FC1, FC2, FC6				
	**124**	Fz, Cz, F3, FC5, FC1, FC2, FC6				
	**128**	Fz, Cz, F3, FC5, FC1				
	**132**	F3, FC5				
	**136**	FC5				
**Effect of case in pseudowords**	**64**	P3	**272**	Pz	**412**	FC6
	**68**	P3	**276**	Pz, Cz	**416**	FC5, FC6
	**72**	FC2, P3	**280**	Pz, Cz, F3, FC1, CP1	**420**	FC6
	**76**	Fz, FC1, FC2, P3	**284**	Pz, Cz, F3, FC5, FC1, CP5, CP1	**424**	FC6
	**80**	Fz, Cz, FC1, FC2	**288**	Cz, F3, FC5, FC1, CP5	**436**	FC6
	**84**	Fz, Cz, FC1, FC2, P3	**292**	Cz, F3, FC5, CP5	**440**	FC6
	**88**	Fz, Pz, Cz, F3, FC1, FC2, FC6, P3	**296**	Cz, F3, FC5	**464**	FC5
	**92**	Fz, Pz, Cz, F3, FC1, FC2, FC6, CP5, CP2, P3, P4	**300**	Cz, F3, FC5	**468**	FC5
	**96**	Fz, Pz, Cz, F3, F4, FC1, FC2, FC6, CP2, P3, P4	**304**	Cz, F3, FC5		
	**100**	Fz, Pz, Cz, F3, F4, FC1, FC2, FC6, P3, P4	**308**	F3, FC5, FC2	**472**	FC5
	**104**	Fz, Pz, Cz, F3, F4, FC1, FC2, FC6, P3	**312**	F3, FC5, FC2	**476**	CP5, P3
	**108**	Fz, Cz, F3, F4, FC1, FC2, FC6	**316**	Fz, F3, FC5, FC1, FC2	**480**	CP5, P3
	**112**	Fz, Cz, F3, F4, FC1, FC2, FC6	**320**	Fz, Cz, F3, FC5, FC1, FC2, FC6	**484**	CP5, CP1, CP2, P3
	**116**	Fz, F3, FC1, FC2, FC6	**324**	Cz, FC5, FC1, FC2, FC6	**488**	CP5, CP1, CP2, P3
	**120**	Fz, F3, FC5, FC1, FC2, FC6	**328**	Cz, FC5, FC2	**492**	CP5, CP1, CP2, P3
	**124**	Fz, F3, FC5, FC1, FC2, FC6, CP5	**340**	FC5, FC2	**496**	CP5, CP1, CP2, P3
	**128**	Fz, F3, FC5, FC1, CP5	**344**	FC5, FC2, CP5	**500**	CP5
	**132**	F3, FC5	**348**	FC5, FC2, CP5		
	**136**	F3, FC5	**352**	FC5, FC2, CP5		
	**140**	F3	**356**	Fz, FC5, FC2, FC6, CP5		
	**144**	F3	**360**	Fz, F3, FC5, FC1, FC2, FC6, CP5		
	**148**	Cz, F3	**364**	Fz, F3, FC5, FC1, FC2, FC6, CP5		
	**152**	Cz, F3, FC5, FC1	**368**	Fz, F3, FC5, FC2, FC6, CP5		
	**156**	Fz, Cz, F3, FC5, FC1, FC2, CP5, CP1	**372**	FC5, CP5		
	**160**	Fz, Cz, F3, F4, FC5, FC1, FC2, CP5				
	**164**	Fz, Cz, F3, F4, FC5, FC1, FC2, CP5				
	**168**	Fz, Cz, F3, FC5, FC1, FC2				
	**172**	Fz, Cz, F3, FC5, FC1				
	**176**	FC5				

Supplementary material: Appendix B. Comparison with previously published ERP data from hearing readers.

In order to test whether the magnitude of the ERP effect of case (difference between the amplitudes in the matched and mismatched case conditions) is the same for deaf and hearing readers, we contrasted the present data with previously published data from hearing readers (Vergara-Martínez et al, 2015). We performed ANOVAs on the size of the effect of case including the within subjects factors hemisphere, A-P distribution and Lexicality (words vs. pseudowords) and the between subjects factor Group (deaf vs. hearing readers). In all analyses, List (1–4) was included as a dummy between-subjects factor. A separate ANOVA was run for each of the identified components (N/P150, N250 and N400), the selected time windows for each of these components was appropriately adapted to the corresponding dataset. For the deaf participants, we used the time windows described above. For hearing participants, the three windows of interest were 80-150, 250-350 and 400-500 ms as in the original article. Results from these analyses are displayed in table 3, the relevant interactions with group are detailed in the text below.

N/P150. There was a significant interaction between A-P distribution and group, $F(1,34) = 7.03$, $p = .012$,: the effect of case was larger for deaf than hearing participants at posterior electrodes ($F = 6.93$, $p = .013$) but not at anterior electrodes ($F <1$).

There was a significant interaction between lexicality and group, $F(1,34) = 4.16$, $p = .049$,: the effect of case was larger for deaf than hearing participants for pseudowords ($F = 9.13$, $p = .005$) but not words ($F < 1$).

N250. There was a significant interaction between A-P distribution and group, $F(1,34) = 6.8$, $p = .013$,: the effect of case was larger for deaf than hearing participants at posterior ($F = 5.6$, $p = .024$) but not at anterior electrodes ($F <1$).

There was a significant interaction between hemisphere, lexicality and group, *F*(1,34) = 4.43, *p* = .043,: the effect of case was larger for deaf than hearing participants for pseudowords at the left hemisphere electrode sites (*F* = 8.64, *p* = .006; remaining *p*s >.1).

N400. There was a significant interaction between hemisphere and group, *F*(1,34) = 6.34, *p* = .017,: the effect of case was larger for deaf than hearing participants at right sites (*F* = 6.25, *p* = .017) but not left hemisphere sites (*F* < 1).

Supplementary table 3. Summary of statistics for the group comparison

		**N/P150**		**N250**		**N400**	
	df	*F*	*p*	*F*	*p*	*F*	*p*
AP	(1,34)	2.88	.099	4.79	.036	6.50	.016
Hem	(1,34)	5.22	.029	19.63	.000	6.13	.018
Lexical	(1,34)	2.04	.163	13.31	.001	3.31	.078
AP * Hem	(1,34)	2.08	.159	15.12	.000	6.80	.013
AP * Lexical	(1,34)	13.54	.001	20.68	.000	14.56	.001
Hem * Lexical	(1,34)	5.70	.023	31.78	.000	7.85	.008
AP * Hem * Lexical	(1,34)	0.89	.353	23.04	.000	8.55	.006
Group	(1,34)	0.70	.409	4.28	.046	1.68	.203
AP * Group	(1,34)	7.03	.012	6.80	.013	2.31	.138
Hem * Group	(1,34)	0.40	.531	1.60	.215	6.34	.017
Lexical * Group	(1,34)	4.16	.049	4.13	.050	3.70	.063
AP * Hem * Group	(1,34)	1.19	.283	1.11	.300	2.85	.100
AP * Lexical * Group	(1,34)	2.85	.101	3.93	.056	0.02	.894
Hem * Lexical * Group	(1,34)	0.05	.833	4.43	.043	0.41	.529
AP * Hem * Lexical * Group	(1,34)	1.28	.267	1.30	.263	3.21	.082

Supplementary material: Appendix C. Repetition priming

This experiment included, as customary, an unrelated priming condition. For comparability with previous research, results from the masked repetition priming effect are presented in this appendix (see Ref. [17] for the same approach). We contrasted word and pseudoword targets preceded by an identity prime (mismatched case) or an unrelated prime (half of the unrelated items in lowercase and half in uppercase).

Behavioural results:

Separate ANOVAs with the within subjects factors Lexicality (Words vs. Pseudowords) and Repetition (Identity vs. Unrelated) were run for the latency and accuracy data. List was included in the analysis as a dummy factor. Incorrect responses (8.7 %) and lexical decision times above and below the 2.5 SDs of the average per participant and condition (1.1 %).

The ANOVA on the latency data showed that response times to words were faster than to pseudowords (716.2 vs. 867.5 ms; $F1(1,16) = 25.78$, $p < .001$; $F2(1,302) = 201.86$, $p < .001$). In addition, targets preceded by a mismatched case identity priming were responded to faster than targets preceded by unrelated primes (766 vs. 817.7 ms; $F1\ (1,16) = 40.25$, $p < .001$; $F2(1,302) = 41.82$, $p < .001$). The interaction between Lexicality and Repetition only approach significance in the subjects' analysis, $F1\ (1,16) = 3.20$, $p = .092$; $F2(1,302) = 4.76$, $p = .006$.

The ANOVA on the accuracy data revealed that participants were more accurate giving a "No" response for pseudowords than a "Yes" response for words (94.3 vs. 88.3 % correct responses respectively; $F1(1,16) = 17.84$, $p = .001$; $F2(1,302) = 3.01$, $p = .083$). Responses to targets preceded by repeated primes were more accurate than those preceded by unrelated primes (93.3 vs. 89.3 % correct responses respectively; $F1(1,16) = 20.62$, $p < .001$; $F2(1,302) = 1.89$, $p = .17$). The interaction between lexicality and repetition was significant $F1(1,16) = 17.18$, $p = .001$; $F2(1,302) < 1$: responses were more accurate for the word targets preceded by repeated primes than for those preceded by unrelated primes (92.1 vs. 84.6 % correct

respectively; $F1(1,16) = 55.95$, $p < .001$; $F2(1,302) = 2.07$, $p = .15$). There were no differences for pseudoword targets ($F1(1,16) < 1$; $F2(1,302) < 1$).

ERP results

Figure 5 (panel b) shows the ERP waves for words and pseudowords preceded by mismatched case identity primes and unrelated primes. After a brief negative going potential peaking around 50 ms, there is a large positive potential peaking around 200 ms. Following this peak, for words only, the repetition condition shows larger positive amplitude values than the unrelated condition. No differences are apparent for pseudowords.

Words and pseudowords were submitted to separate mass univariate analysis analogous to those of the main comparison in this study (repeated measures, two-tailed t-tests at each sampling point between 50 and 550 ms at 15 scalp electrodes, using an FDR level of 5%). Results for word targets showed significant repetition priming effects sporadically between 160 and 168 ms at anterior left electrodes. Widely distributed differences were found between 200 and 304 ms and finally, differences in posterior electrodes were found between 380 and 488 ms. No differences were found for pseudoword targets. That is, we found a repetition priming effect on the N250 and N400 components for word but not for pseudoword targets.

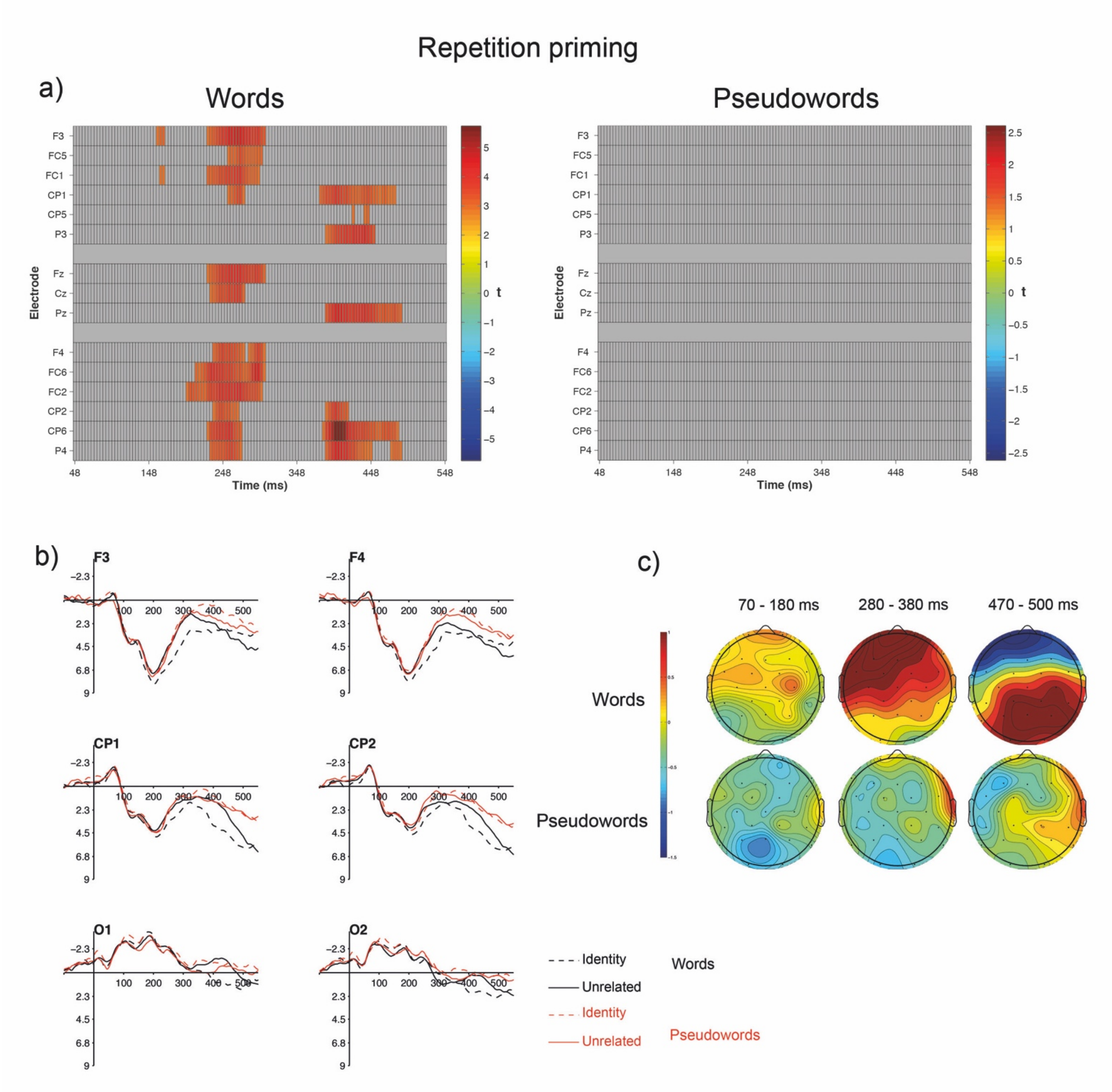

Supplementary figure S5. ERP results. Panel a) shows the results of the mass univariate statistical analysis of the time course of the repetition priming effect for words (left) and pseudowords (right) targets separately. The plots convey the results of the comparisons between 50 and 550 ms at the 15 electrodes analysed. T values are colour coded according to the legend shown at the right for each of the comparisons. Panel b) shows the ERP waves at 6 representative electrodes for the conditions of interest. Panel c) shows the scalpmaps for time windows equivalent to the analysed in the main text.

Supplementary table 4. Detailed results of the Mass Univariate Analyses of the repetition effect.

Analysis parameters			
Time boundaries of 50 to 550 ms for hypothesis test (exact window boundaries 48 to 548 ms).	Number of channels: 15. Number of time points: 126. Total # of comparisons: 1890	Number of participants: 20. t-score degrees of freedom: 19	FDR control procedure: Benjamini & Hochberg (independent or positive dependency)

ERP component Comparison	N/P150 ms	N/P150 electrodes with significant differences	N250 ms	N250 electrodes with significant differences	N400 ms	N400 electrodes with significant differences
**Repetition priming in words**	**160**	F3	**200**	FC2	**380**	CP1
	**164**	F3, FC1	**204**	FC2	**384**	CP1, CP6
	**168**	F3, FC1	**208**	FC2	**388**	Pz, CP1, CP2, CP6, P3, P4
			**212**	FC2, FC6	**392**	Pz, CP1, CP2, CP6, P3, P4
			**216**	FC2, FC6	**396**	Pz, CP1, CP2, CP6, P3, P4
			**220**	FC2, FC6	**400**	Pz, CP1, CP2, CP6, P3, P4
			**224**	FC2, FC6	**404**	Pz, CP1, CP2, CP6, P3, P4
			**228**	Fz, F3, FC1, FC2, FC6, CP6	**408**	Pz, CP1, CP2, CP6, P3, P4
			**232**	Fz, Cz, F3, FC1, FC2, FC6, CP6, P4	**412**	Pz, CP1, CP2, CP6, P3, P4
			**236**	Fz, Cz, F3, F4, FC1, FC2, FC6, CP2, CP6, P4	**416**	Pz, CP1, CP2, CP6, P3, P4
			**240**	Fz, Cz, F3, F4, FC1, FC2, FC6, CP2, CP6, P4	**420**	Pz, CP1, CP6, P3, P4
			**244**	Fz, Cz, F3, F4, FC1, FC2, FC6, CP2, CP6, P4	**424**	Pz, CP5, CP1, CP6, P3, P4
			**248**	Fz, Cz, F3, F4, FC1, FC2, FC6, CP2, CP6, P4	**428**	Pz, CP1, CP6, P3, P4
			**252**	Fz, Cz, F3, F4, FC1, FC2, FC6, CP2, CP6, P4	**432**	Pz, CP1, CP6, P3, P4
			**256**	Fz, Cz, F3, F4, FC5, FC1, FC2, FC6, CP1, CP2, CP6, P4	**436**	Pz, CP1, CP6, P3, P4
			**260**	Fz, Cz, F3, F4, FC5, FC1, FC2, FC6, CP1, CP2, CP6, P4	**440**	Pz, CP5, CP1, CP6, P3, P4
			**264**	Fz, Cz, F3, F4, FC5, FC1, FC2, FC6, CP1, CP2, CP6, P4	**444**	Pz, CP5, CP1, CP6, P3, P4
			**268**	Fz, Cz, F3, F4, FC5, FC1, FC2, FC6, CP1, CP2, CP6, P4	**448**	Pz, CP1, CP6, P3, P4
			**272**	Fz, Cz, F3, F4, FC5, FC1, FC2, FC6, CP1, CP6, P4	**452**	Pz, CP1, CP6, P3
			**276**	Fz, Cz, F3, F4, FC5, FC1, FC2, FC6, CP1	**456**	Pz, CP1, CP6
			**280**	Fz, F3, FC5, FC1, FC2, FC6	**460**	Pz, CP1, CP6
			**284**	Fz, F3, F4, FC5, FC1, FC2, FC6	**464**	Pz, CP1, CP6
			**288**	Fz, F3, F4, FC5, FC1, FC2, FC6	**468**	Pz, CP1, CP6
			**292**	Fz, F3, F4, FC5, FC1, FC2, FC6	**472**	Pz, CP1, CP6
			**296**	Fz, F3, F4, FC5, FC1, FC2, FC6	**476**	Pz, CP1, CP6, P4
			**300**	Fz, F3, F4, FC5, FC2, FC6	**480**	Pz, CP1, CP6, P4
			**304**	Fz, F3, F4, FC6	**484**	Pz, CP6, P4
					**488**	Pz, P4
**Repetition priming in Pseudowords**	ERPs are NOT significantly different from 0.000000 (q=0.050000) at any time point/window analysed.					

Correlations with reading-related measures.

No correlations were found between the size of the behavioural effect of Case for pseudowords and performance in any of the reading-related measures (all *p*s > .05). The size of the effect for words correlated significantly with Sentence reading (r = .46, *p* = .043) but not with Reading comprehension (r = .17, *p* = .48) not orthographic/visual bias during syllabification index (r = .22, *p* = .37).
